# A Case Report of Inflammatory Myofibroblastic Tumor: A Rare Benign Lung Tumor

**DOI:** 10.7759/cureus.59237

**Published:** 2024-04-28

**Authors:** Amr M Allama, Ghaidaa A Almuhammadi, Rawia A Alzughaibi, Raha Z Ishqi, Mohammed A Al-Refai

**Affiliations:** 1 Thoracic Surgery, King Fahad General Hospital, Madinah, SAU; 2 College of Medicine, Taibah University, Madinah, SAU

**Keywords:** pseudotumor, inflammatory pseudotumor, inflammatory myofibroblastic tumor, benign tumor, lung tumors

## Abstract

Inflammatory myofibroblastic tumors (IMTs) of the lung are a rare type of mesenchymal tumors that tend to occur more in the lungs of children. They are extremely rare in adults. IMTs require extensive pulmonary resection because they are commonly locally invasive. The key to preventing recurrence is complete resection, and the prognosis is excellent after surgery. We report a case of a patient with an inflammatory pseudotumor of the lung. The patient is a 27-year-old female who presented with a dry cough. A chest radiograph and computed tomography showed a lesion in the left main bronchus and near-total left lung collapse. As surgery was necessary to establish the diagnosis, left pneumonectomy was performed followed by a histological examination of the surgical specimen which confirmed inflammatory pseudotumor.

## Introduction

Lung inflammatory myofibroblastic tumors (IMT) often arise as a result of excessive inflammation. It is one of the rare benign lung tumors [1-3]. This tumor commonly arises in the lungs, although it can develop in other regions of the body [4]. It accounts for 0.7% of all lung tumors. First described by Brunn in 1939, it is an inflammatory, reactive, and non-neoplastic process characterized by the unregulated growth of inflammatory cells. However, it is not a reaction process. It is a true tumor and is typically discovered incidentally and most frequently affects children and young people [5,6]. Several nonspecific clinical and radiological manifestations are present. Without a surgical excision, the diagnosis is difficult to establish [2,7].

## Case presentation

A 27-year-old female, without significant medical history or history of smoking, presented to the emergency room with severe hypoxia and cyanosis with oxygen saturation of 45% in room air. She complained of a persistent, nonproductive cough, described as dry and associated with shortness of breath, which had been present for approximately three months with a gradual onset and progressive course. There were no signs of hemoptysis, weight loss, fever, and night sweating.

**Figure 1:**
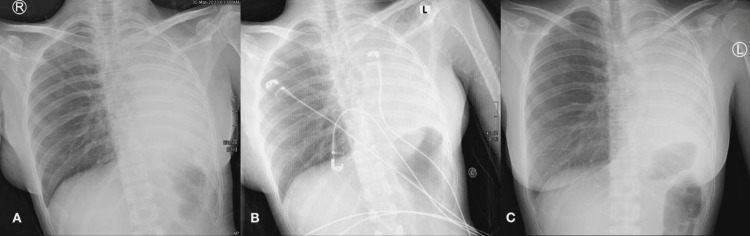
Chest X-ray showing complete left lung collapse.

**Figure 2:**
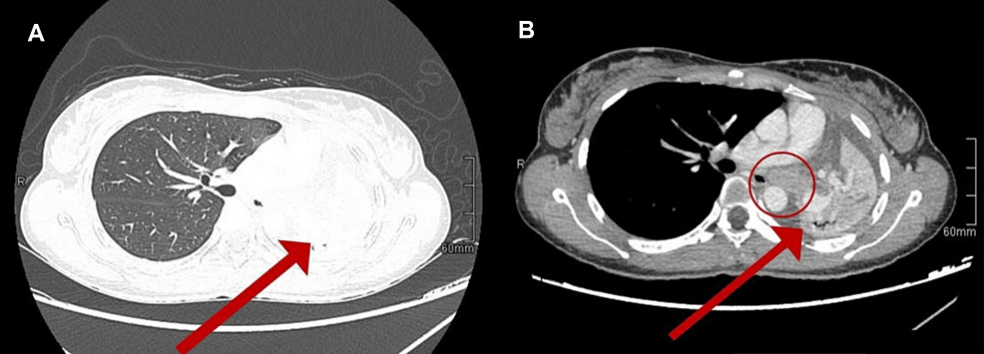
Axial chest CT without contrast (A) and with contrast (B) showing near total lung collapse (red arrow) showing lesions 30x19 mm (red circle) in the left main bronchus with bronchial obstruction

She was admitted to the intensive care unit and put on mechanical ventilation for five days. Chest examination revealed that the trachea was slightly deviated to the left, with reduced chest movement on the left side, dullness on percussion, and an absent breath sound on the left side. All the other systems were essentially normal. Laboratory values were significant for white blood cell count of 15.5×10^9^/L, neutrophilic count of 12.1×10^9^ /L, lymphocytic count of 1.5×10^9^ /L, monocytic count of 1.5×10^9^ /L, and lymphocytic percentage of 8.8. The results of tumor markers were negative; all other laboratory values were unremarkable. A chest x-ray showed a complete left lung collapse with the trachea slightly deviated to the left (Figure 1). A CT scan of the chest was done, showing near-total left lung collapse with shifting of the mediastinum toward the left side and mild left pleural effusion (Figure 2).

A soft tissue lesion (30×19 mm) in the left hilum was found, causing complete attenuation of the left major stem bronchus and a few solid nodules in the lateral segment of the right lower lobe. There was no other mass effect on vascular hilar structures. CT scans of the abdomen and pelvis show no sign of metastasis. Bronchoscopy showed a complete obstruction of the left main bronchus, and bronchial lavage was done. A positron emission tomography (PET) scan was not performed due to unavailability.

**Figure 3:**
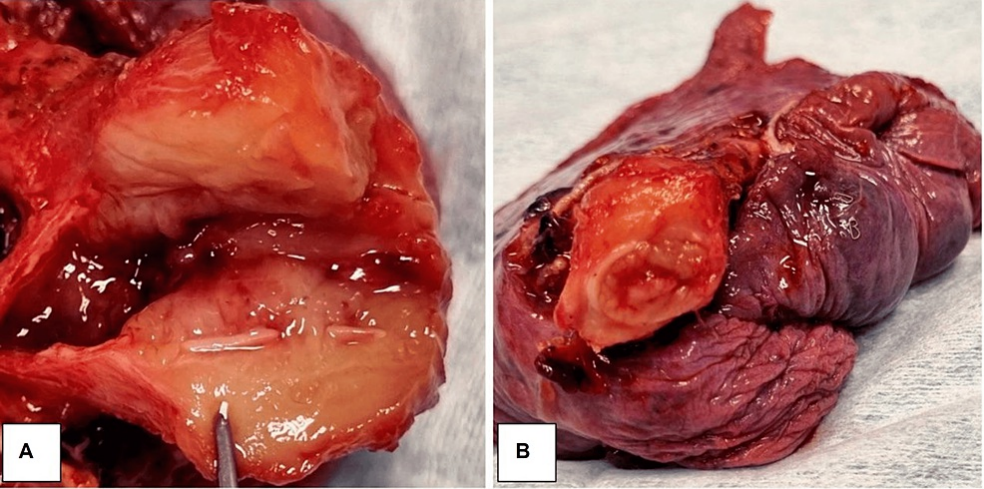
Resected gross specimen of the lung with inflammatory myofibroblast tumor

**Figure 4:**
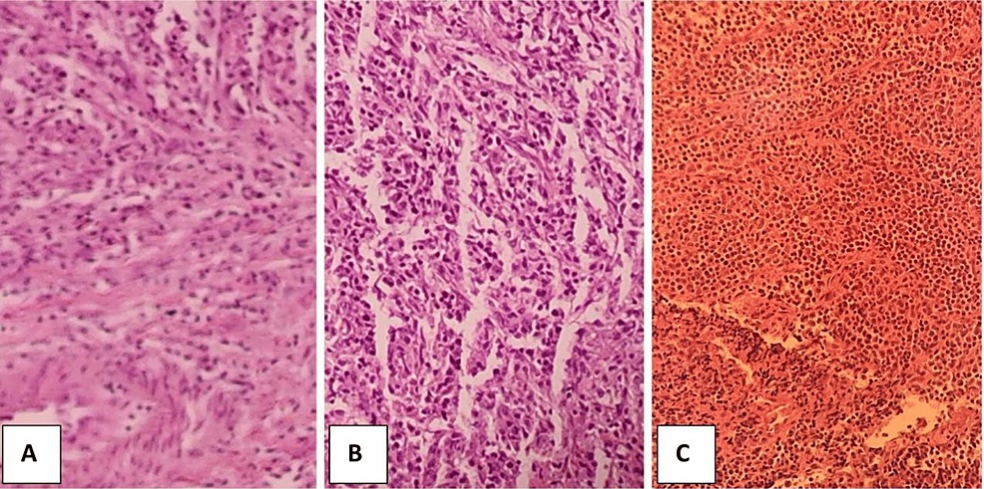
Histological images (hematoxylin & eosin, x100)

**Figure 5:**
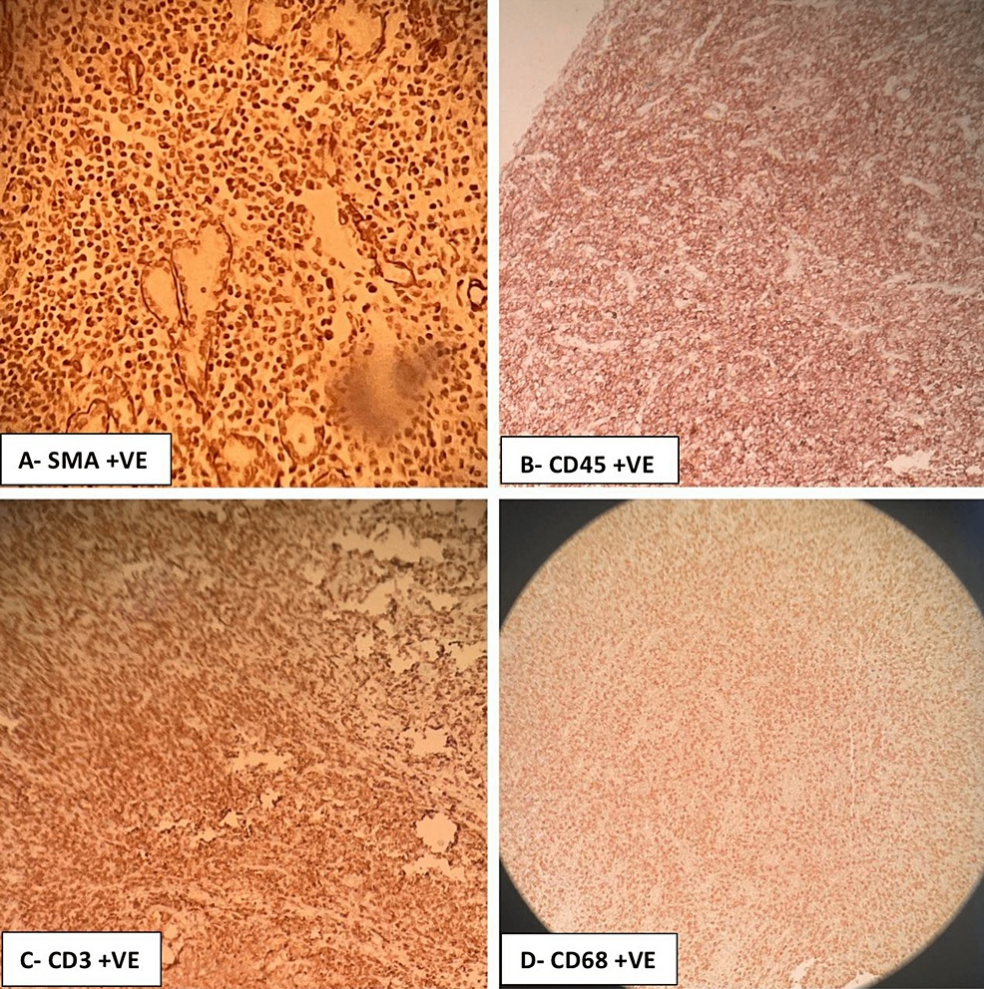
Immunohistochemical analysis (x100) showing positive staining for SMA, CD45, CD3, CD68 SMA: smooth muscle actin

**Figure 6:**
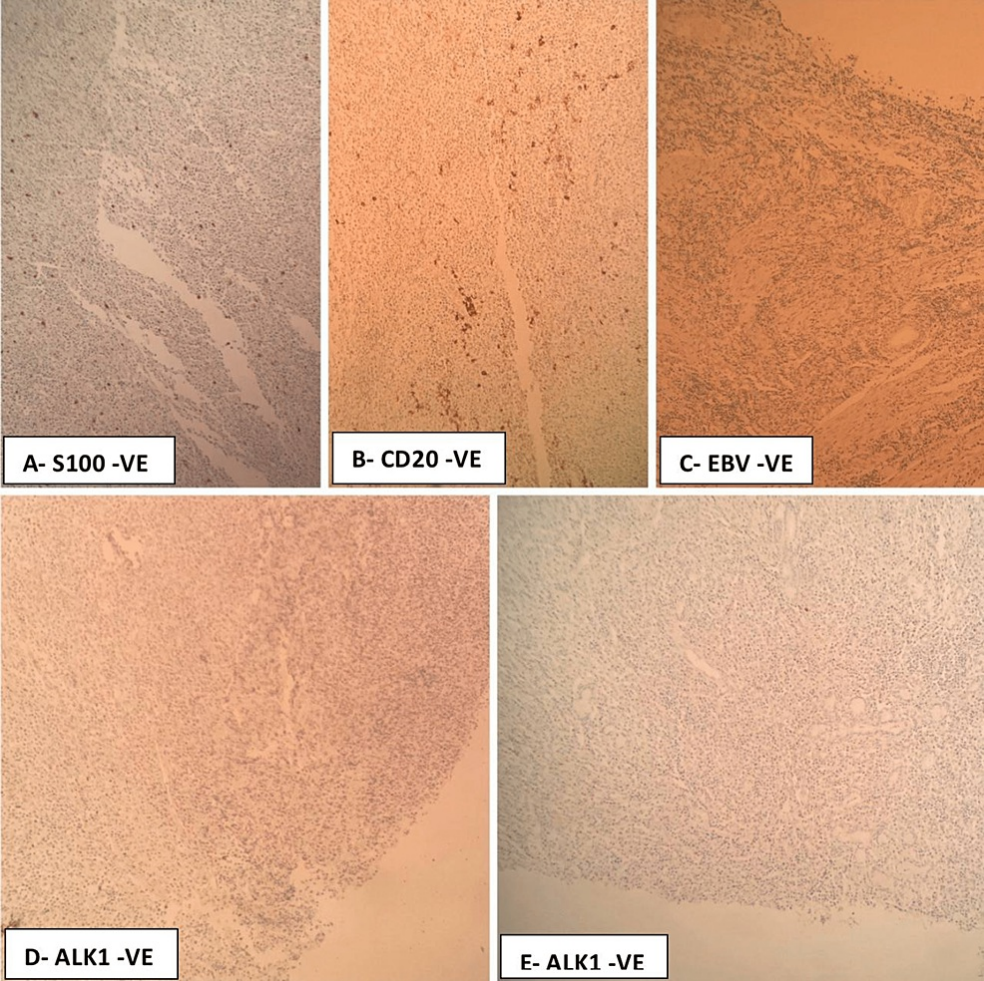
Immunohistochemical analysis (x100) showing negative for ALK1, S100, CD20, EBV EBV: Epstein-Barr virus

After 13 days, the patient underwent a left-side pneumonectomy. Gross examination of the surgical specimen showed that the tumor was 3 cm in size, soft in consistency, and white in color (Figure 3). Microscopic examination (Figure 4) showed predominantly fibrohistiocytic/fibroinflammatory cells with numerous histiocytes, lymphocytes, and myofibroblasts with plasma cells, and mitosis was less than 3/50 high power field (HPF). Focal necrosis, areas of disintegrated muscle, and collagenous bundles were noted. Hyperplasia in mucinous glands was seen with scattered inflammatory cell infiltrates. Moreover, there was inflammation encasing the bronchus and peribronchial tracheal rings. Bronchiectasis and intra-alveolar hemorrhage were also reported. Immunohistochemical analysis showed positive staining for SMA, CD45, CD3, and CD68 (Figure 5). In contrast, the tumor cells were not reactive for ALK1, CKPAN, S100, CD20, or EBV (Figure 6).

Post-operative evaluation was done, symptoms improved, and the patient was discharged two weeks later. Three months after surgery, she presented to the clinic with no complaints, and a chest CT scan was performed revealing no evidence of tumor recurrence.

## Discussion

Inflammatory pseudotumors of the lung are rare and were first described in 1939 [5]. This tumor is also known as a plasma cell granuloma. It used to be categorized as a benign tumor. However, it can be recurrent at the same site [8,9]. Less than 1% of lung tumors in adults are IMTs, despite the fact that they make up 20-50% of all primary lung tumors in children [10,11]. They are not only found in the lungs but can arise in the brain, liver, spleen, lymph nodes, salivary glands, breast, soft tissues, and skin [8]. The most common sites are the lung, abdominopelvic region, and retroperitoneum. Commonly, this tumor only affects one organ but can occur in multiple organs [9]. The pathophysiology and etiology are still unknown. There are various theories, most of which postulate an exaggerated immunologic response to a viral or foreign antigen-antibody reaction [6]. The main cell type is the myofibroblast, a cell involved in tissue repair, and it often contains varying levels of stromal and cellular components [12].

Diverse nomenclature has been used to describe these lesions, including inflammatory pseudotumor, inflammatory myofibroblastic proliferation, inflammatory fibrosarcoma, xanthogranuloma, plasma cell histiocytoma complex, plasma cell granuloma, and fibrous histiocytoma [9,13]. The etiology and pathogenesis remain uncertain [8]. Many patients have inflammatory pseudotumors that were accidentally found on a chest radiograph while they were asymptomatic [14]. In about 70% of cases, the disease is accidentally detected on imaging scans requested for another cause [15]. Patients may have symptoms like fatigue, fever, hemoptysis, shortness of breath, chest pain, and coughing. These symptoms are determined by the tumor's size and location [10,16]. In about 90% of the cases, radiographic findings revealed a solitary peripheral lung nodule. Lower lobes, peripheral lung parenchyma, and subpleural locations are preferred [16]. The lesion presents as a heterogeneous mass with contrast enhancement on computed tomography. Calcifications, cavitations, and lymphadenopathy are uncommon [9].

PET can be useful to differentiate benign IMT from malignant lesions [9]. Bronchoscopy and fine needle aspiration biopsy samples are frequently small and insufficient for simultaneous diagnosis [9]. Therefore, the preferred diagnostic method is surgical removal of the lesion [8]. Histological analysis revealed various myofibroblastic cells arranged in a myxoid, fibrous, or calcified stroma, along with a component of chronic inflammation distributed to varying degrees throughout the tumor. There have been three established histological patterns. The first is myxoid and richly vascularized and has the appearance of nodular fasciitis or granulation tissue. The second pattern is a more dense spindle cell proliferation with focal nodular lymphoid hyperplasia resembling fibromatosis. The third type is that of a very sclera-hyalinized, slightly cellular stroma [17]. Because of the diverse nature of the tissue of origin, most IMTs immunostain positive for anaplastic lymphoma kinase-1 (ALK-1), caldesmon, desmin, and often for keratin cocktail, smooth muscle actin (SMA), and S100. Immunohistochemical staining is useful to differentiate IMTs from tumors with the same histopathology, and expression of ALK-1 is highly specific for IMTs [8], but in our case, ALK-1 was negative. Locally invasive, recurring, and metastatic IMTs present issues of malignant illness, whereas locally limited disease detected early is amenable to complete resection with a benign prognosis. Small tumors and tumors that can be completely resected have a better prognosis and survival rate. Only 2% of patients experience recurrence after complete resection versus 60% after incomplete resection [10,18].

When surgery is not an option or the tumor is multilocular, pharmacologic treatment (such as glucocorticoids and chemotherapy) or radiotherapy may be considered appropriate. Recurrent illness is still a possibility even years after the first diagnosis. After resection, patients should be closely followed in order to detect local or distant recurrence [17].

## Conclusions

Pulmonary IMT is a rare benign tumor. Clinical and radiological findings are not specific. The diagnosis can only be confirmed with a histopathological study. Despite being a benign tumor, it warrants a complete surgical resection due to its ability for recurrence and local invasion. When it's possible, complete resection is safe and provides a high survival rate. It's the best way to prevent recurrence.

## References

[1] Demirhan O, Ozkara S, Yaman M, Kaynak K (2013). A rare benign tumor of the lung: Inflammatory myofibroblastic tumor - case report. Respir Med Case Rep.

[2] Takeda S, Onishi Y, Kawamura T, Maeda H (2008). Clinical spectrum of pulmonary inflammatory myofibroblastic tumor. Interact Cardiovasc Thorac Surg.

[3] Hammas N, Chbani L, Rami M (2012). A rare tumor of the lung: inflammatory myofibroblastic tumor. Diagn Pathol.

[4] Tan-Liu NS, Matsubara O, Grillo HC, Mark EJ (1989). Invasive fibrous tumor of the tracheobronchial tree: clinical and pathologic study of seven cases. Hum Pathol.

[5] Ochs K, Hoksch B, Frey U, Schmid RA (2010). Inflammatory myofibroblastic tumour of the lung in a five-year-old girl. Interact Cardiovasc Thorac Surg.

[6] Zhang Y, Dong ZJ, Zhi XY, Liu L, Hu M (2009). Inflammatory myofibroblastic tumor in lung with osteopulmonary arthropathy. Chin Med J (Eng).

[7] Chen CK, Jan CI, Tsai JS (2010). Inflammatory myofibroblastic tumor of the lung--a case report. J Cardiothorac Surg.

[8] Marwah N, Bhutani N, Dahiya S, Sen R (2018). Inflammatory pseudotumour: a rare tumor of lung. Ann Med Surg (Lond).

[9] Na YS, Park SG (2018). Inflammatory myofibroblastic tumor of the pleura with adjacent chest wall invasion and metastasis to the kidney: a case report. J Med Case Rep.

[10] Sagar AE, Jimenez CA, Shannon VR (2018). Clinical and histopathologic correlates and management strategies for inflammatory myofibroblastic tumor of the lung. A case series and review of the literature. Med Oncol.

[11] Barbetakis N, Efstathiou A, Xenikakis T, Konstantinidis H, Fessatidis I (2006). An unusual cause of haemoptysis in a young male. Int Semin Surg Oncol.

[12] Dehner LP (2000). The enigmatic inflammatory pseudotumours: the current state of our understanding, or misunderstanding. J Pathol.

[13] Surabhi VR, Chua S, Patel RP, Takahashi N, Lalwani N, Prasad SR (2016). Inflammatory myofibroblastic tumors: current update. Radiol Clin North Am.

[14] Copin G (1996). Plasma cell granuloma of the lung: difficulties in diagnosis and prognosis. Ann Thorac Surg.

[15] Al-Obaidi A, Buess C, Mogire J, Reddy PS (2019). Inflammatory myofibroblastic tumor of the lung: an extremely rare condition in adults. Cureus.

[16] Elmadi A, Rami M, Khattala K (2011). Inflammatory pseudotumors of the lung in a child. J Pediatrie Pueric.

[17] Braham Y, Migaou A, Njima M (2020). Inflammatory myofibroblastic tumor of the lung: A rare entity. Respir Med Case Rep.

[18] Melloni G, Carretta A, Ciriaco P (2005). Inflammatory pseudotumor of the lung in adults. Ann Thorac Surg.

